# A Case of Iatrogenic Right-to-Left Shunting after Atrial Septal Defect Closure

**DOI:** 10.70352/scrj.cr.25-0329

**Published:** 2025-09-09

**Authors:** Naohiko Oki, Naritaka Kimura, Hideaki Shimizu, Hideyuki Shimizu

**Affiliations:** Department of Cardiovascular Surgery, Keio University School of Medicine, Tokyo, Japan

**Keywords:** atrial septal defect, right-to-left shunting, adult congenital heart disease, eustachian valve

## Abstract

**INTRODUCTION:**

The main causes of right-to-left shunting (RLS) in patients with atrial septal defect (ASD) are pulmonary hypertension, right ventricular outflow tract obstruction, severe tricuspid regurgitation, and a large ASD resulting in equal bi-atrial pressures. Reports of a case of an unintentional connection of the inferior vena cava (IVC) to the left atrium (LA) discovered many years after the repair of ASD are rare.

**CASE PRESENTATION:**

A 47-year-old male with a history of congenital ASD repair was found to have large RLS during examination of choledocholithiasis. Details of the former surgeries, performed twice for some reason, were unknown. He had cyanosis of fingers, but neither pulmonary hypertension nor right ventricular outflow obstruction. Transesophageal echocardiography, cardiac CT, and cardiac catheterization demonstrated a direct connection between the IVC and the LA as well as a residual ASD. The patient subsequently underwent successful surgical repair. It was speculated that the eustachian valve (EV) of the IVC had been wrongly taken as the lower margin of the defect in the first repair, and then a new ASD was created in the second surgery to maintain his hemodynamics. Though he had RLS with ASD, he subsequently lived a normal life for approximately 40 years, albeit with exertional dyspnea and mild cyanosis.

**CONCLUSIONS:**

We report on a case of iatrogenic RLS after ASD closure. The surgeon must always check the intracardiac anatomy carefully and close the ASD without using other structures such as the EV.

## Abbreviations

ASD
: atrial septal defect
EV
: eustachian valve
IVC
: inferior vena cava
LA
: left atrium
RA
: right atrium
RLS
: right-to-left shunting
TEE
: transesophageal echocardiography

## INTRODUCTION

The main causes of right-to-left shunting (RLS) in patients with atrial septal defect (ASD) are pulmonary hypertension, right ventricular outflow tract obstruction, severe tricuspid regurgitation, and a large ASD resulting in equal bi-atrial pressures.^1,2)^ Reports of a case of an unintentional connection of the inferior vena cava (IVC) to the left atrium (LA) discovered many years after the repair of ASD are rare. We report a case of an adult patient with ASD who suffered from major iatrogenic RLS following a previous first surgery, after which a new ASD was created during a second surgery to improve the physiological circulatory abnormality. He subsequently lived a normal life for approximately 40 years, albeit with exertional dyspnea and mild cyanosis.

## CASE PRESENTATION

**Fig. 1:**
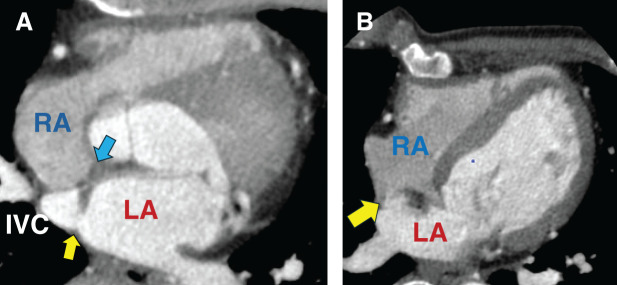
Preoperative CT. (**A**) Venous blood from the inferior vena cava flows into the left atrium (yellow arrow) instead of the right atrium. Eustachian valve (blue arrow). (**B**) New ASD (yellow arrow) created in the second surgery. ASD, atrial septal defect; IVC, inferior vena cava; LA, left atrium; RA, right atrium

A 47-year-old male with a history of congenital ASD, initially diagnosed and surgically repaired at age 3, was referred to our department for evaluation of cyanosis of the fingers during examination of choledocholithiasis. The heart surgeries had been performed twice previously, but the details were unknown. He had no heart murmur and no symptoms in daily life other than exertional dyspnea and mild cyanosis. His hemoglobin level was 15.5 g/dL. The arterial oxygen saturation was 91% on room air, and he had clubbed fingers on both hands. He had no history of thromboembolism or renal dysfunction. Chest X-ray demonstrated no cardiac enlargement. Transesophageal echocardiography (TEE) demonstrated a residual ASD and no connection between the IVC and the right atrium (RA). Cardiac CT demonstrated a direct connection between the IVC and the LA, as well as the residual ASD (**Fig. 1**). At the time of preoperative cardiac catheterization, the catheter passed directly from IVC to LA and then to the RA, showing the connection between the IVC and the LA, and the residual ASD with mainly left-to-right shunting. Pulmonary arterial pressure was within the normal range.

**Fig. 2:**
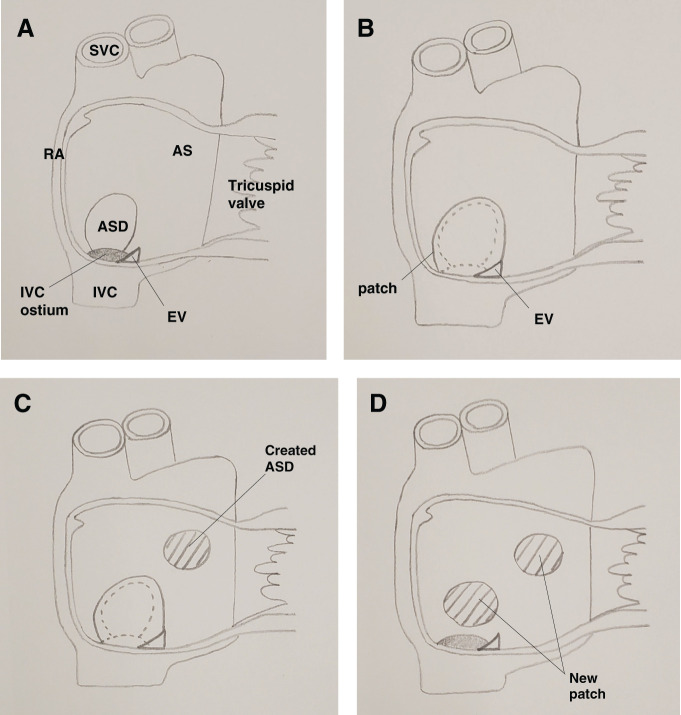
Schema of intracardiac structure. (**A**) Original anatomy; large EV was near the ASD. (**B**) In the first surgery, the EV had been wrongly taken as the lower margin of the ASD. (**C**) In the second surgery, the new ASD was created in the AS. (**D**) In the last surgery, the previous patch including EV was completely resected and defects were closed with a bovine pericardial patch in the correct position. AS, atrial septum; ASD, atrial septal defect; EV, eustachian valve; IVC, inferior vena cava; RA, right atrium; SVC, superior vena cava

The patient subsequently underwent surgical repair of the residual ASD and rerouting of IVC flow to the RA with a bovine pericardial patch. It was speculated that the eustachian valve (EV) of the IVC had been wrongly taken as the lower margin of the defect in the first surgery (**Fig. 2A** and **2B**), and that a new ASD was created in the second surgery to maintain his hemodynamics (**Fig. 2C**). During the surgery at this time, the previous atrial septum, including EV, was resected, and a new septum was made from a bovine pericardial patch in the correct position. IVC flow then returned correctly to the RA (**Fig. 2D**). The postoperative course was uneventful, and the cyanosis of his fingers was resolved after surgery.

## DISCUSSION

This patient had normal pulmonary pressure and no right ventricular hypertrophy before surgery. In this case, the cause of RLS was the direct connection between the IVC and the LA, which might have been iatrogenically created during the previous surgery.

The EV is a remnant of the fetal IVC valve and is typically found at the junction of the IVC and the RA.^2,3)^ The EV of the IVC might have been wrongly taken as the lower margin of the defect in the initial repair of the ASD, which caused a large RLS. There are some case reports that an EV itself naturally causes RLS in patients with ASD.^4,5)^ An EV increases RLS in patients with ASD by directing IVC flow into the LA. However, in this case, the past surgery was the main cause of the RLS. What is more, though it is a speculation, the patient may have suffered from severe hypoxia after the first surgery, and then a new ASD was created to improve hypoxia in the second surgery. After that, this patient could live his daily life for a long time, albeit with exertional dyspnea and mild cyanosis. The left-to-right shunt ratio of the new ASD was 1.62, and there was no right-to-left shunt of the new ASD just before the surgery. If the patient had sufficient examinations, such as cardiac catheterization and TEE, when he suffered from severe hypoxia, he could have received the correct surgery. There are some reports describing the iatrogenic diversion of blood from the IVC to the LA during ASD repair in patients with lower margin defects, resulting in severe cyanosis and hypoxia.^1,6)^ Our case was probably the same situation, and a thorough investigation should have been conducted to determine why severe cyanosis and hypoxia occurred at the time. A prominent EV encroaching on the lower portion of the atrial septum could easily be misconstrued as the inferior rim of the ASD.^1)^ The surgeon must always keep in mind the possibility that the inferior edge of the defect may be the EV. If the error occurs, it is necessary to reoperate as soon as possible, and the new patch for the atrial septum should be replaced in the correct position so that the orifice of the IVC opens into the RA.^6)^ There is no doubt that perioperative TEE can be helpful in evaluating the morphology of the defect and the EV in patients with ASD.^1)^

It has been reported that partial diversion of the IVC to the LA would present with delayed cyanosis and hypoxia.^7)^ Even if the patient does not have severe cyanosis or hypoxia postoperatively, patients should be monitored carefully in the outpatient clinic. What is more, the relief of pulmonary venous congestion and right ventricular strain by the correction of the left-to-right shunt, the occurrence of only partial diversion of the IVC flow to the LA, and the occurrence of stenosis of the IVC with collaterals draining to the superior vena cava through an azygos vein may mask the discovery of iatrogenic diversion of IVC flow to the LA, which can cause cyanosis and hypoxia.^7)^ Although it is a speculation that a new ASD was created to maintain his hemodynamics in the second surgery, this masked the discovery of iatrogenic diversion of IVC flow to the LA.

## CONCLUSIONS

We report a case of iatrogenic RLS after ASD closure. The surgeon must always check the intracardiac anatomy carefully and close the ASD without using other structures such as the EV.
